# Supplementary figures and images for: Genome-wide identification, structural and gene expression analysis of the nitrate transporters (NRTs) family in potato (Solanum tuberosum L.)
Source: PLoS One. 2021 Oct 21;16(10):e0257383. doi: 10.1371/journal.pone.0257383 (PMC8530285; doi:10.1371/journal.pone.0257383)

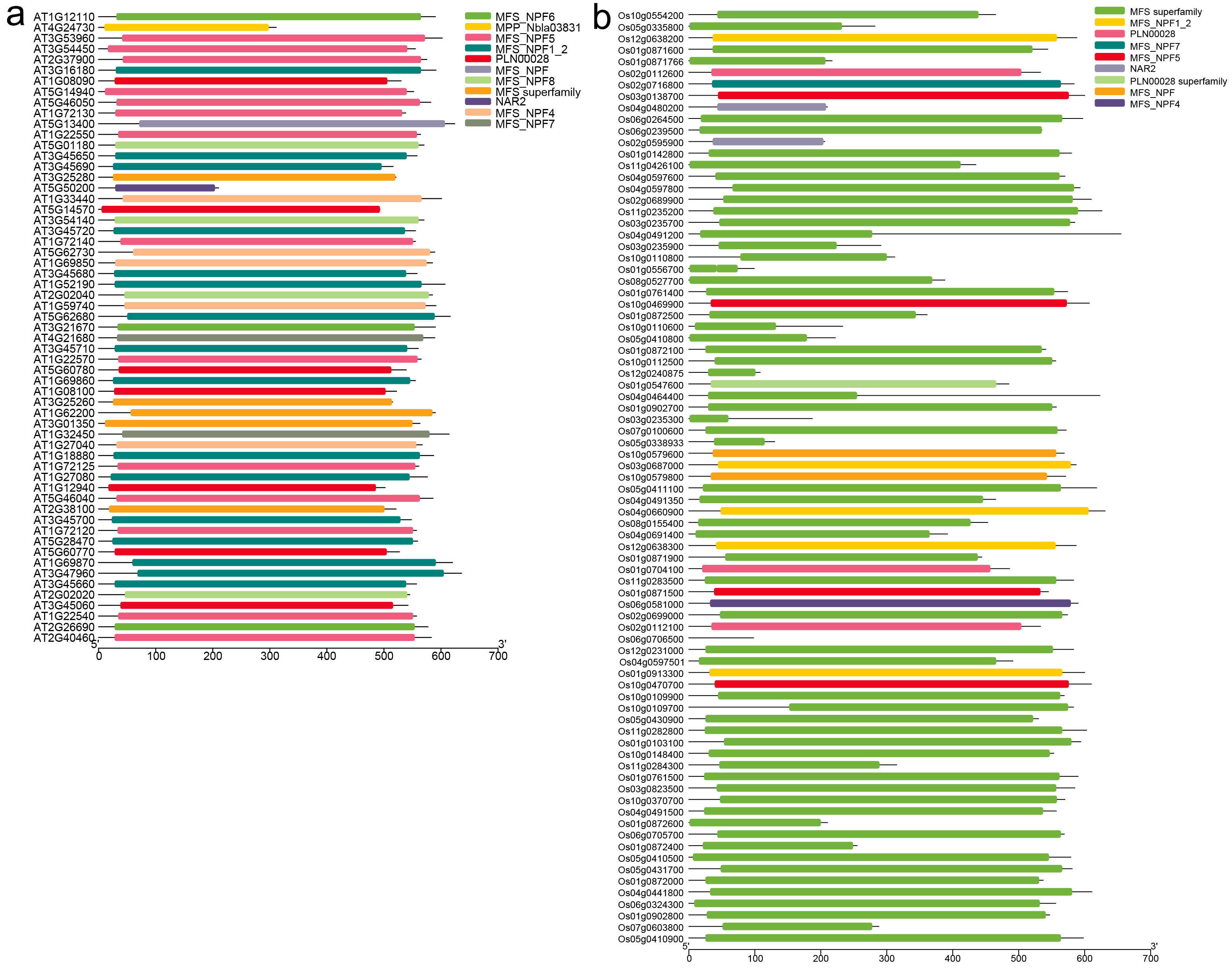

Supplement: S1 Fig — (a) and (b) shows the Conserved domain identification of NRT genes in Arabidopsis thaliana and rice, respectively. The abscissa represents the amino acid length. Different colors represent different domains. (TIF) [file pone.0257383.s001.tif]

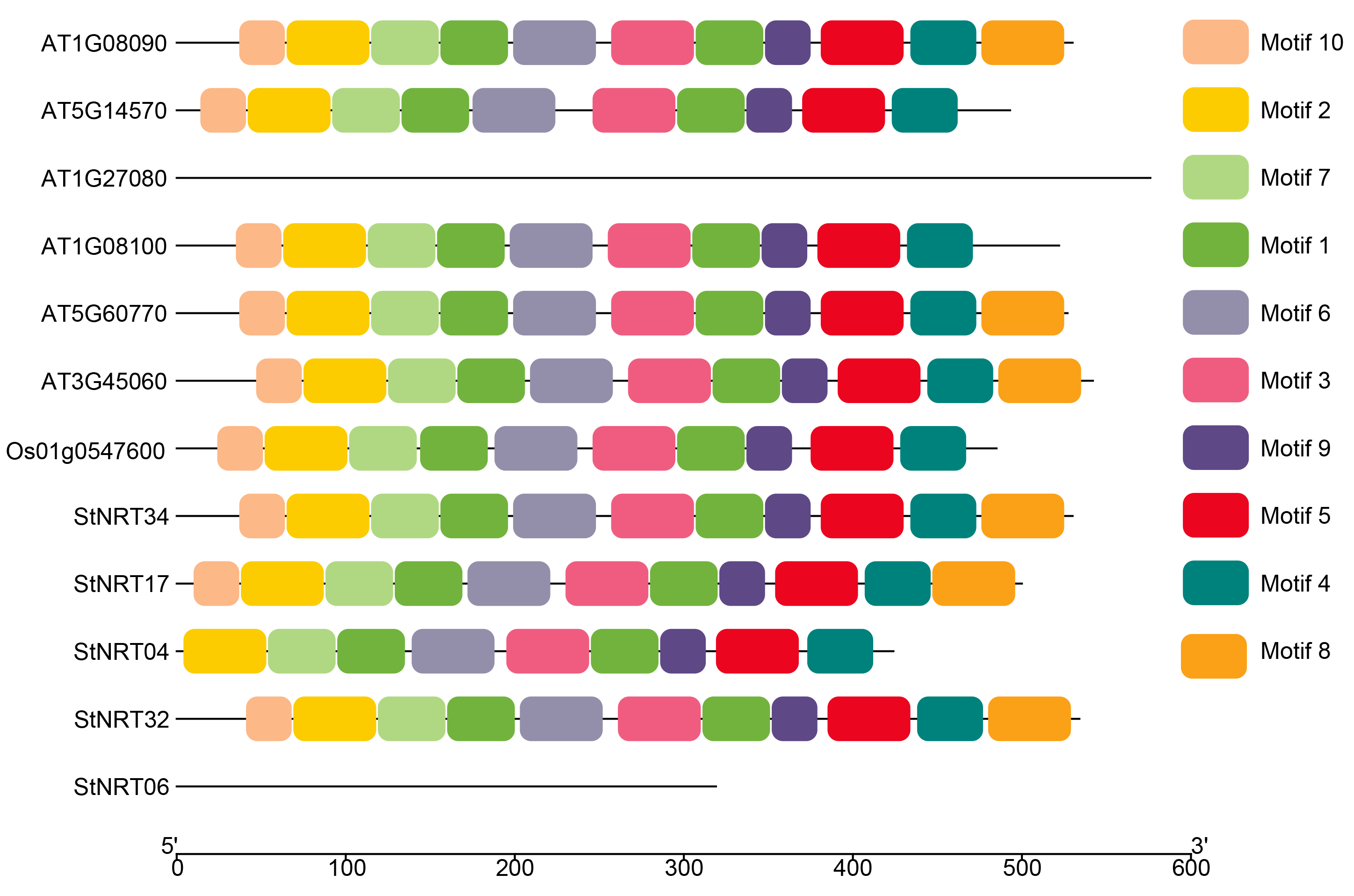

Supplement: S2 Fig — Distributions of conserved motifs in StNRT members. Ten putative motifs are indicated in different colored boxes. (TIF) [file pone.0257383.s002.tif]
