# Supplementary material for: Genome-wide identification, structural and gene expression analysis of the nitrate transporters (NRTs) family in potato (Solanum tuberosum L.)
Source: PLoS One. 2021 Oct 21;16(10):e0257383. doi: 10.1371/journal.pone.0257383 (PMC8530285; doi:10.1371/journal.pone.0257383)
Supplement: Attachment — Submitted filename: Comments.docx [file pone.0257383.s003.docx]

Comments

**Line 56-63.** The authors are using NRT as well as NTR for referring same gene family. This needs clarification.

**Line 65 and Line 288:** whatever little is known about potato NRT should be mentioned in introduction, to explain the relevance and novelty of research. The under-mentioned references are a few that are relevant and demand due citation in this MS

Tiwari et al., 2020, Plant Physiol Biochem, 154:171-183

Zhang et al., 2020 PLOS ONE 15(10): e0240662

Pieczynski et al.. 2017, Plant Biotech j 16:603-614

**Line 85:** The authors should have also included solanacae family also to get a better overview

**Line 139-141**: The SRA ID given cannot be retrieved from NCBI, if the authors have performed any RNA seq analysis that should be published first to make the data more authentic

**Line 165:** Consistently the mistake of not italicising *Arabidopsis thaliana* is done. This is a serious concern it authors warrant publications in prestigious journals like Plos One.

**Line 188:** Same mistake repeated. It questions the scientific writing skill of the authors.

**Line 202:** suddenly CIPK gene is mentioned without any prior abbreviations or relevance.

**Line 219**: Explanation for mentioning PLN00028 domain suddenly is required.

**Line 328:** very vague justification is supplemented.

**Line 342:**  what is the meaning of TFs, it has been used without prior citation.

**Discussion:** The section has been prepared with least effort with improper and insufficient references and justification. The importance, relevance, uniqueness of the work is not reflected. Corroboration with previous studies is also not mentioned and contradictory results not justified properly.
