# Supplementary material for: Genome-wide identification, structural and gene expression analysis of the nitrate transporters (NRTs) family in potato (Solanum tuberosum L.)
Source: PLoS One. 2021 Oct 21;16(10):e0257383. doi: 10.1371/journal.pone.0257383 (PMC8530285; doi:10.1371/journal.pone.0257383)
Supplement: Attachment — Submitted filename: response.docx [file pone.0257383.s004.docx]

Dear Editor and reviewers,

Thank you for your letter and for the reviewers’ comments concerning our manuscript entitled “Genome-wide identification, structural and gene expression analysis of the nitrate transporters (NRTs) family in potato (Solanum tuberosum L.)”. Those comments are all valuable. We have studied comments carefully and made corrections.

The revised manuscript is highlighted in Tracked Changes version.

Reviewer #1: Comments
Line 56-63. The authors are using NRT as well as NTR for referring same gene family. This needs clarification.

Response: We have unified the abbreviation of the full text. Thank you for your comment.

Line 65 and Line 288: whatever little is known about potato NRT should be mentioned in introduction, to explain the relevance and novelty of research. The under-mentioned references are a few that are relevant and demand due citation in this MS
Tiwari et al., 2020, Plant Physiol Biochem, 154:171-183
Zhang et al., 2020 PLOS ONE 15(10): e0240662
Pieczynski et al.. 2017, Plant Biotech j 16:603-614

Response: Thank you for your comment. We have revised this part according to your suggestions. The sentence in the abstract and line-288 are also deleted.

Line 85: The authors should have also included solanacae family also to get a better overview

Response: Thank you for your comment. Your opinions and suggestions are very valuable and worth our attention. However, the aim of this study was to screen the expression levels of *NRT* gene family members in potato under efficient nitrogen conditions, and to explore the effect of nitrogen deficiency on *SRTs*. Adding data of other Solanaceae plants can show the evolutionary relationship of *NRT* gene among different species, which is not consistent with the purpose of this study. But it is indeed a good research direction. Thank you again for your advice.

Line 139-141: The SRA ID given cannot be retrieved from NCBI, if the authors have performed any RNA seq analysis that should be published first to make the data more authentic

Response: Thank you for your comment. Here, we rephrase it to reduce the reader's misunderstanding. This data is uploaded from our previous research

Line 165: Consistently the mistake of not italicising Arabidopsis thaliana is done. This is a serious concern it authors warrant publications in prestigious journals like Plos One.

Response: Thank you for your reminding. We have revised it as required.

Line 188: Same mistake repeated. It questions the scientific writing skill of the authors.

Response: Done as required.

Line 202: suddenly CIPK gene is mentioned without any prior abbreviations or relevance.

Response: Dorry for the basic mistakes. It should be *StNRT*.

Line 219: Explanation for mentioning PLN00028 domain suddenly is required.

Response: Thank you for your comment. We have added the explanation for PLN00028.

Line 328: very vague justification is supplemented.

Response: Thank you for your comment. We cited our previous references for discussion. According to our previous study, the NRT transcripts showed different expression profiles in different potato breeds, especially for varieties with different sensitivity to N deficiency [22]. Hence, we inferred that this might be due to the genetic differences in different potato breeds.

Line 342: what is the meaning of TFs, it has been used without prior citation.

Response: Dorry for the basic mistakes. TFs is short for transcription factors

Discussion: The section has been prepared with least effort with improper and insufficient references and justification. The importance, relevance, uniqueness of the work is not reflected. Corroboration with previous studies is also not mentioned and contradictory results not justified properly.

Response: Thank you for your comment. In this round of revision, we have made major changes to the discussion part. We hope this revision will improve the quality of the manuscript.

Reviewer #2: Authors have identified 39 StNRT gene family members the potato genome. The subfamily classification, gene structure and distribution analysis, and conserved domain prediction for StNRT genes were performed using various bioinformatics tools. Authors downloaded the publicly available RNA seq data and discussed the tissue specific and N2 specific expression of StNRT genes.

Minor comments:

1.Line 24: Nitrate (NO3¯) is the most important source of mineral N for plant growth (rewrite [Nitrogen (N2)]

2. Line 32: For the expression, 33 Different (different) members of StNRT play different roles in leaves and roots.

3. Line 34: different members have a clear distribution in different 35 organizations. (organs?)

4. Line 35: And most of the StNRTs might be regulated by TFs like MYB, MYC and 36 ERE. (rewrite, remove and)

5. Line 45: different organizations. (organs/tissues)

6. Line 35: (organs/tissues)

7. Line 62: Remove reference as text: Dechorgnat et al reported that 62 there were three NTR gene subfamilies: NRT1, NRT2, and NRT3 [16].

8. Line 95: To identified (identify) the domains of the candidate members,
9. Line 102: All the candidate StNRTs were mapped on potato chromosome (chromosomes)
10. Line 104: To identified (identify)
11. Line 105: comparison of all StNRT members were (was) carried
12. rearrange Column 1 in table 1. Make the gene name in one row.
13. Elaborate the Table 1 legend.
14. Elaborate the figure 1 legend.
15. Line 199: Rewrite: Figure 3 showed the location of all these 39 StNRT members, which were 200 randomly distributed on the 12 potato chromosomes.
16. Line 202: Why authors discussed about CIPK or it is just StNRT (clarify), StNRT genes, while chromosome 03 and 06 contains most CIPK genes (5 in each)
17. Line 209: Figure 3 Chromosomal location and gene duplication (mention species, gene family)
18. Line 215: software. The results showed that 10 motifs were identified (Figure 4a). Rewrite
19. Kine 220: we performed motif analysis base on genes of Arabidopsis thaliana (Rewrite)
20. Line 240: Rewrite: we found that all StNRT members in all potato had orthologous genes in
21. Line 227. Figure 4(.) Conserved motif and gene structure analysis
22. Line 245 Figure 5 (.) Collinearity analysis of StNRT members.
23. Line259-261: The expression of StNRT24, 260 StNRT14, StNRT22, StNRT26, StNRT20, StNRT10, StNRT09 and StNRT03 is 261 relatively high. (Which tissue or condition)
24. Line 266: The redder the color, the higher the expression; the bluer the color, the 267 lower the expression. (Write according to previous literature) follow the color range in box.
25. Line 278: Figure 7(.) Predicted cis-elements in StNRT promoters
26. Line 290: Lezhneva reported that there were 24 AtNRTs in Arabidopsis 291 [18] and Li identified 48 candidate NRT genes in pineapple [21] (Remove the named citations and rewrite the sentence)

27. Line 301-302: genes were closely related to each other and had not yet differentiated into fully differentiated gene subfamilies. (use another appropriate word).

Response: Thank you very much for your careful review. We are sorry for the basic mistakes we made. We accept your suggestion and have made point-to-point modifications to the manuscript. We hope this revision can improve the quality of the manuscript.

Major comments:
1. Check English formatting, grammatical errors.

Response: Thank you for your comment. We invited our international student Vigo to edit the English, which improved the readability of this manuscript.

2. The details and proper condition/treatments of samples used in RNA seq is missing.

Response: Thank you for your comment. This data is uploaded from our previous research. We cited this article in the place of the data source. In addition, we described the design in the results section and the legend in Figure 6.

3. Verify the tissue specific expression pattern of some differentially expressed StNRT genes, also confirm the N2 specificity of StNRT genes by wet lab experiments.

Response: Thank you for your comment. Due to sample reasons, qRT-PCR experiments cannot be performed. But according to this previously published article [1], qRT-PCR data and RNA-seq data were in excellent consistency, so we believe that this RNA-seq data is reliable.

[1]. Zhang J, Wang Y, Zhao Y, Zhang Y, Zhang J, Ma H, et al. Transcriptome analysis reveals Nitrogen deficiency induced alterations in leaf and root of three cultivars of potato (Solanum tuberosum L.). PLOS ONE. 2020;15(10):e0240662. doi: 10.1371/journal.pone.0240662.
