# Supplementary material for: Genome-wide identification, structural and gene expression analysis of the nitrate transporters (NRTs) family in potato (Solanum tuberosum L.)
Source: PLoS One. 2021 Oct 21;16(10):e0257383. doi: 10.1371/journal.pone.0257383 (PMC8530285; doi:10.1371/journal.pone.0257383)
Supplement: Attachment — Submitted filename: response.docx [file pone.0257383.s005.docx]

Dear Editor and reviewers,

Thank you for your letter and for the reviewers’ comments concerning our manuscript entitled “Genome-wide identification, structural and gene expression analysis of the nitrate transporters (NRTs) family in potato (Solanum tuberosum L.)”. Those comments are all valuable. We have studied comments carefully and made corrections.

The revised manuscript is highlighted in Tracked Changes version.

Reviewer #1: Comments

**Comments:**

Prior to the acceptance, the authors needs to rectify the MS for grammatical and scientific errors, like use of botanical names at all places and missing italics at some places. In the abstract the authors have mentioned, evolutionary relationship as a scope of the study, however, in the justification to reviewers, they have mentioned that evolutionary studies is beyond the scope of the study. This needs to be rectified.
**Response:** thank you for your comments. First of all, thank you for your recognition. We further revised the graphical and scientific errors as required. Also, we revised the scope in the abstract section.

**Comments:**

line no. 29-30: proper meaning is not reflected.

**Response:** thank you for your comments. We rephased the sentence to “Totally, 39 StNRT gene members were identified in potato genome, including 33, 4 and 2 member belong to NRT1, NRT2, and NRT3, respectively.”

**Comments:**

Table 1: use pI; PI is not the proper notion.
line 204: MEGA 7.0, incorrectly written

**Response:** thank you for your comments. Both the two errors were revised as required.

Reviewer #2: Comments

**Comments:**

Authors have carefully edited the manuscript and addressed all previous concerns. Authors should proof read the manuscript one more time before publishing.

**Response:** Thank you for your recognition. We have revised the grammatical and scientific errors of the manuscript. Thank you again for your review.
